# Clinical Response and Transfusion Reactions of Sheep Subjected to Single Homologous Blood Transfusion

**DOI:** 10.1155/2014/734397

**Published:** 2014-12-03

**Authors:** Rejane Santos Sousa, Antonio Humberto Hamad Minervino, Carolina Akiko Sato Cabral Araújo, Frederico Augusto Mazzocca Lopes Rodrigues, Francisco Leonardo Costa Oliveira, Clara Satsuki Mori, Janaina Larissa Rodrigues Zaminhan, Thiago Rocha Moreira, Isadora Karolina Freitas Sousa, Enrico Lippi Ortolani, Raimundo Alves Barrêto Júnior

**Affiliations:** ^1^Department of Clinical Science, Faculty of Veterinary Medicine, University of Sao Paulo, Avenida Professor Orlando Marques de Paiva, 87 Cidade Universitária, 05508-270 São Paulo, SP, Brazil; ^2^Institute of Biodiversity and Forest, Federal University of Western Pará, Avenida Vera Paz S/N, Salé, 68000-000 Santarém, PA, Brazil; ^3^Department of Animal Science, Federal Rural University of the Semiarid Region, Avenida Francisco Mota, s/n Bairro Presidente Costa e Silva, 59625-900 Mossoró, RN, Brazil

## Abstract

Studies in relation to blood conservation and responses to transfusion are scarce for ruminants. We evaluated the clinical manifestations of sheep that received a single homologous transfusion of whole blood, focusing on transfusion reactions. Eighteen adult sheep were subjected to a single phlebotomy to withdraw 40% of the total blood volume, which was placed into CPDA-1 bags and then divided into G0, animals that received fresh blood, and G15 and G35, animals that received blood stored for 15 or 35 days, respectively. Clinical observations were recorded throughout the transfusion, whereas heart rate, respiratory rate, and rectal temperature were assessed at the following times: 24 hours after phlebotomy and before transfusion; 30 minutes, six, twelve, 24, 48, 72, and 96 hours and eight and 16 days after transfusion. All groups presented transfusion reactions, among which hyperthermia was the most frequent (50% of animals). Tachycardia occurred most frequently in the G35 animals (50% of them). During transfusion G35 animals presented more clinical manifestation (*P* < 0.05). Transfusion of fresh or stored total blood improved the blood volume, but transfusion reactions occurred, demonstrating that a single transfusion of fresh or stored blood can cause inflammatory and febrile nonhemolytic transfusion reactions in sheep.

## 1. Introduction

Studies in the field of hemotherapy for ruminants are scarce, particularly in relation to blood conservation and responses to transfusion. Even through the illnesses that require blood transfusion are known, this therapeutic measure is sometimes used without proper criteria and without considering the potential risks of transfusion reactions [1, 2].

Although blood storage is an important evolution in the field of transfusion, stored red blood cells undergo a series of changes known as storage lesions, which tend to become worse with the passage of time [3].

Sousa et al. [4] stored total blood from sheep in blood bags containing a conserving solution of citrate, phosphate, dextrose, and adenine (CPDA-1) for 35 days and found that there were progressive increases in the concentrations of plasma hemoglobin, potassium, and lactate, and decreases in blood pH and in the concentrations of sodium, bicarbonate, and glucose. These storage lesions are indicative of loss of quality of the stored blood, which may contribute towards occurrences of post-transfusion reactions [5, 6].

Post-transfusion reactions are defined as any adverse effect after receipt of total blood or blood components [1]. Hunt and Moore [7] considered that the commonest signs of transfusion reactions in ruminants are tachycardia, tachypnea, sudoresis, tremors, fever, pruritus, dyspnea, hematuria, and hemoglobinuria, among others. However, some reports claim that transfusion reactions in ruminants receiving a single blood transfusion are uncommon, as these animals have low levels of circulating isoantibodies [7, 8].

The main nonfatal transfusion reactions described in the literature are febrile nonhemolytic transfusion reactions (FNHTRs), which are divided into nonsymptomatic (hyperthermia, due to an increase in temperature ≥ 1°C, without other symptoms) and symptomatic. Symptomatic FNHTRs are classified as inflammatory (presence of hot and cold flushes, tremors or stiffness, with or without accompanying hyperthermia) or allergic (presence of erythema, rubor, or urticaria, among others) [9]. Other types of transfusion reaction are such as hemolysis, circulatory overload, and anaphylaxis [9].

Thus, the present study aimed to evaluate the clinical response and the occurrence of transfusion reactions in sheep receiving a single homologous transfusion of blood either fresh or stored for two different periods.

## 2. Material and Methods

The present study was approved by the Bioethics Committee for Animal Use (CEUA) of Federal Rural University of the Semiarid Region (Protocol number 31/2011). Eighteen healthy sheep (12 females and 6 males) aged three to four were used. They were hybrids of the Santa Inês breed, with mean weight 52.89 kg ± 6.67 kg. They were kept in collective pens, separated according to sex, and underwent a 30-day adaptation period. During this period, they received an application of endectocide based on moxidectin (Cydectin, Fort Dodge), vaccine against clostridiosis (Covexin 10, Schering-Plough), and doses of coccidiostats (Coccifin, Ouro Fino). All sheep underwent weekly parasitological examinations to rule out the presence of endoparasites during the entire experimental period. The animals' baseline diet was calculated on the basis of 2.3% of their live weight and was composed of 75% dry material (hay from coast-cross grass) and 25% commercial concentrated feed, which was supplied twice a day. The sheep also received complete mineral supplements every day (Ovinofós, Tortuga) (15 g/animal) and they had free access to water. None of the animals were previously submitted to any kind of blood transfusion.

Acute anemia was induced in all the 18 sheep by withdrawing 40% of their blood volume, measured according to the calculation that the animals' total volume of blood corresponded to 8% of their body weight [10]. Twenty-four hours after induction of anemia, the animals were divided into three experimental groups: G0 (control group, *n* = 6) received a transfusion of fresh blood that had been collected in CPDA-bags (collected just prior transfusion); G15 (15-day group, *n* = 6) received a transfusion of total blood that had been stored in CPDA-1 bags for 15 days; and G35 (35-day group, *n* = 6) received a transfusion of total blood that had been stored in CPDA-1 bags for 35 days. Each experimental group was composed of four females and two males. The blood donor and recipient were always of the same sex and had similar body weights. Before blood transfusion a cross-matching blood compatibility test was performed between donor and receiver [11].

The blood transfused into the animals of G15 and G35 was stored in a refrigerator at a temperature ranging from 2 to 4°C. For the transfusion blood bags were removed from the refrigerator and after 30 minutes in room temperature the blood was transfused to the animals. Each animal received 20 mL of total blood per kg of body weight during the transfusion. Over the first 30 minutes of the transfusion, the blood was infused at the rate of 0.25 mL/kg; after this period, the rate was increased to 5 mL of blood per kg of body weight per hour. During the experimental period the ambient temperature ranged from 22 to 28°C.

Clinical observations were documented throughout the transfusion, whereas heart rate, respiratory rate and rectal temperature were evaluated at the following times: 24 hours after phlebotomy and before transfusion (*T*0); 30 minutes after transfusion (*T*30 m); 6, 12, 24, 48, 72, and 96 hours after transfusion (*T*6, *T*12, *T*24, *T*48, *T*72, and *T*96, resp.); and 8 and 16 days after transfusion (*T*8 d and *T*16 d, resp.). The heart and respiratory rates were measured with the aid of a phonendoscope over a one-minute period, while temperatures were measured using a clinical digital thermometer according to Pugh [12]. In those same times blood sampling was performed for packed cell volume, red blood cell count, and total hemoglobin concentration, which were performed in Automatic hematology analyzer (model 2800 BC Vet, Mindray, Shenzhen, China).

The data from clinical observations over the course of the transfusion and from the individual physical examinations at the abovementioned times were analyzed in conjunction to determine whether any transfusion reactions had occurred. The following were considered to be transfusion reactions: hyperthermia, characterized as a rise in body temperature by 1°C or more, in relation to the baseline temperature; tachycardia, characterized as an increase in heart rate of up to 20% in relation to *T*0; inflammatory reactions, characterized by fasciculation or muscle tremors; and allergic reactions, characterized by the presence of urticaria, erythema, or eruptions on the skin.

The statistical analysis on heart rate, respiratory rate and rectal temperature was performed by means of two-way repeated-measurement analysis of variance (ANOVA), followed by Bonferroni's mean comparison test. The different experimental groups were compared at the different times, and the variables at different times were compared with the baseline values (*T*0). To evaluate the occurrence of clinical manifestation during the transfusion in the different groups a *χ* ^2^ analysis was performed. The significance level was taken to be 5%. The clinical observations and posttransfusion reactions were evaluated by means of descriptive statistics, and the animals were assessed individually over the course of time.

## 3. Results

Table 1 presents the mean values and the standards deviations of the packed cell volume, red blood cells, and total hemoglobin concentration from the three experimental groups during the study.

All the clinical observations during the transfusion period are laid out in Table 2. Considering the *χ* ^2^ analysis between the groups and the occurrence of abnormal clinical observations (tachypnea, fasciculation, sudoresis, bloat, and ejaculation) during the blood transfusion, the G35 animals presented more clinical alterations in comparison with G0 (*P* = 0.010). Only the animals in G35 presented sudoresis, mild tympanism, and ejaculation during the transfusion.

Sheep from all groups defecated and urinated during the transfusion. Among the animals in G0 and G35, defecation and micturition were observed only once in each group, whereas in G15, three animals had more than two occurrences of micturition during the transfusion period.

Considering the evaluation in the moments after the transfusion, three animals from G35 presented tachycardia: one at *T*30 m and two at *T*6. One animal in G0 presented heart rate changes within the first half hour and one animal in G15 at *T*48.

Regarding the presence of hyperthermia, G0 had four cases, among which two sheep had hyperthermia at *T*30 m, and one of these animals continued to present high temperature until *T*96. Two other animals presented temperature rises more than 96 hours after transfusion. Two animals in G15 and G35 presented hyperthermia: one in G15 showed a rise in temperature at *T*30 m that continued until *T*96, while one in G35 continued to have high temperature until *T*24. Another animal in G15 and another in G35 had hyperthermia at *T*72 and *T*24, respectively. Concomitantly with hyperthermia, one animal in G15 and another in G35 had inflammatory transfusion reactions. No allergic reactions to transfusion were detected.

**Figure 1:**
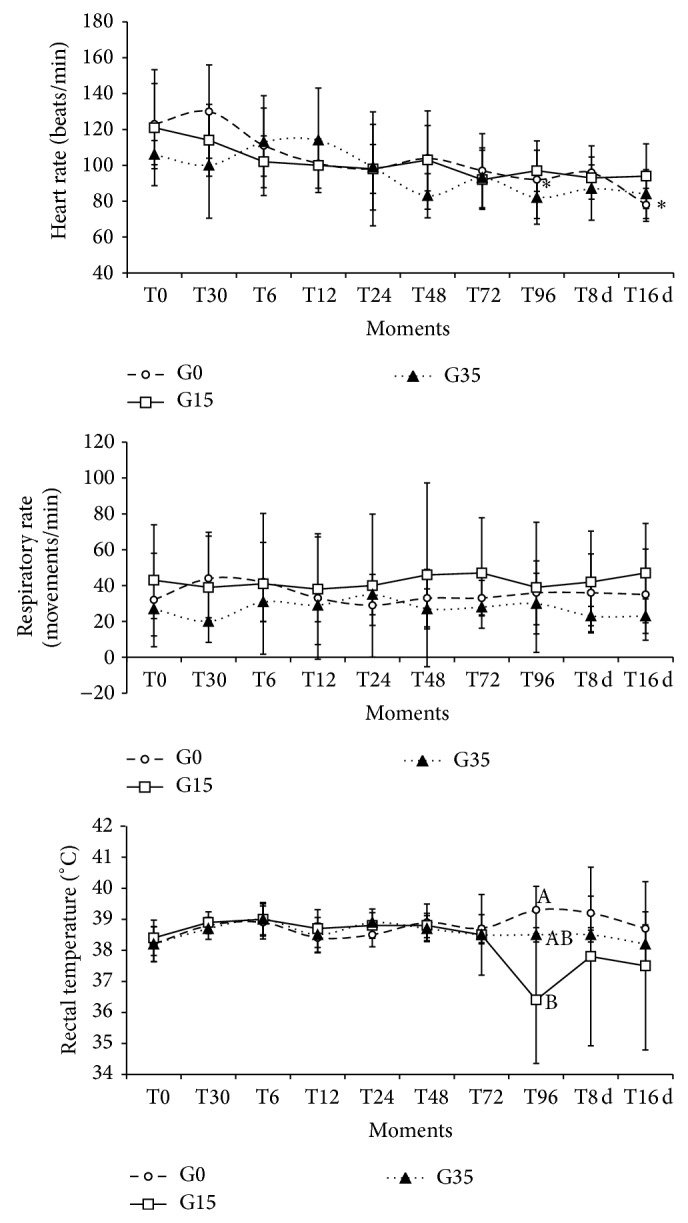
Mean and standard deviation of the heart rate (beats/min), respiratory rate (breaths/minute), and rectal temperature (°C) of sheep after transfusion with whole blood fresh or stored for 15 or 35 days at the time points analyzed. ∗ Mean statistical difference in comparison with *T*0 (*P* < 0.05) in each group. Different capital letters mean difference between experimental groups in one specific moment (*P* < 0.05).

Figure 1 presents the mean values and standard deviations for heart rate, respiratory rate and rectal temperature. There were no significant differences between the three groups in relation to heart rate and respiratory rate. In comparing between the times, there was a reduction in G0 (*P* < 0.05) in heart rate at *T*96 and *T*16 d when compared to *T*0. The mean temperature in G0 was greater than G15 (*P* < 0.05) at *T*96.

## 4. Discussion

Over the last few years, the benefits of blood transfusion have been questioned because of the connection between this practice and adverse effects. In this study we chose to use homologous blood transfusion, because it is the most common form of transfusion for ruminants and the literature is unanimous in stating that ruminants receiving a first transfusion rarely present transfusion reactions [7, 8, 10]. However for sheep, our clinical practice has shown that animals receiving a first transfusion can exhibit clinical changes, even with the compatibility test was performed before transfusion.

From the clinical observations during the transfusion period, micturition and defecation were the most frequent findings in all the experimental groups. This was probably related to the increase in blood volume resulting from the blood infusion, given that in cases of acute anemia, the kidneys are the main organs that suffer vasoconstriction and hypoxia, since the blood is directed towards vital organs such as the brain, heart, and lungs [13, 14]. In situations of acute blood loss or hypovolemia, another compensatory mechanism that the organism has is to diminish its urine production in an attempt to maintain a greater quantity of fluid in the blood vessels [14]. After the transfusion, the increased volumes of fluid in the vessels stimulate vasodilation and the kidneys begin to perform their functions better and, in turn, stimulate diuresis.

The gastrointestinal blood flow also becomes lower in cases of major blood loss, with sympathetic vasoconstriction of the large-caliber intestinal and mesenteric veins. This decreases the blood volume in these veins and thus diverts large quantities of blood to other parts of the circulation [15]. In this manner, after the transfusion, the blood volume becomes redistributed, thereby allowing the gastrointestinal system to resume its normal functioning. This explains why the animals defecated when they started to receive the transfusion.

Tachypnea during the transfusion period was observed in three animals, and one animal in G35 presented concomitant sudoresis. These changes were observed at the start of the transfusion and the situation was resolved by reducing the infusion rate. These symptoms may be associated with the infusion rate [16]. The mild tympanism that was observed in one animal in G35 was linked to the length of time for which the animal remained in decubitus for the transfusion to be performed (two hours and 35 minutes). Ejaculation was observed in only one animal, and it cannot be determined whether this occurrence was connected only with the transfusion event, given that this animal was used for reproduction as a semen donor and was therefore more susceptible to this type of occurrence.

In this study, considering the animals individually, temperature increases were observed in all three experimental groups, and these rises occurred within the first 30 minutes after the transfusion. A rise of 1°C or more in the posttransfusion temperature, in relation to the baseline, was taken to be a parameter for determining whether a FNHTR occurred [9, 17]. The presence of bioreactive substances released by leukocytes (pyrogenic cytokines) during the storage period in the cases of G15 and G35, or the presence of alloantibodies in the receptor that started to act against antigens present in the donor's platelets, red blood cells, or leukocytes in the case of G0, may have contributed towards these reactions [18, 19]. The formation of alloantibodies can occur because previous sensitization due to the transmission of erythrocytes between individuals, which may happen due to general practices such as vaccination, drugs administration or blood sampling without needle exchange or even by bites from ticks or flies [20].

FNHTRs is a reaction by the organism to the presence of cytokines, either through increased production of these mediators by leukocytes during blood storage, or through formation of immune complexes (antigens and antibodies) between antigens of the receptors and cytotoxic antibodies of the donor, thereby stimulating complementary action and production of pyrogenic cytokines (IL-6, TNF-*α*, and IL-1*β*) [21].

FNHTRs in humans may be associated with the presence of hot and cold flushes and stiffness. This type of reaction is not considered to be dangerous for the patient, but occurrences of hemolytic or septic reactions that might also cause fever, hot and cold flushes, and tremors should be investigated [22]. The animals in G15 and G35 presented mild fasciculation during the transfusion, which is indicative of a transfusion reaction of inflammatory effect. These same animals had concomitant hyperthermia, thus suggesting that the length of storage might have contributed towards the occurrence of these reactions. In these cases, when pyrogenic cytokines produced by leukocytes are transfused to the receptor, they stimulate the thermoregulatory center and induce synthesis of prostaglandin E2, which promotes changes to the thermostatic equilibrium. In this manner, the organism is stimulated to produce heat by means of vasoconstriction and muscle movements (hot and cold flushes, tremors, and fasciculation) until the body temperature reaches a new equilibrium point [9, 23]. In humans and dogs, FNHTRs may be accompanied by other symptoms such as nausea, vomiting, hypotension, and dyspnea [2, 21].

In the present study, 67% of the animals in G0 presented hyperthermia, while 33.5% of the animals in G15 and G35 presented elevated temperatures. The hyperthermia observed in G0 may be linked to the presence of alloantibodies formed by previous pregnancies, by the contact of maternal blood and the newborn during birth, stimulating primary production of alloantibodies, since only females showed hyperthermia. Thus, when the animal was transfused (second sensitization) a secondary immune response capable of releasing inflammatory mediators alloantibodies occurred [24].

Storage and alloantibodies may be involved in the hyperthermia occurred in G15 and G35, as both males and females had such alteration. Although we have not determined the concentrations of reactive substances during storage and even after transfusion, studies have shown that leukocytes during storage can produce reactive substances or release inflammatory mediators during lysis [25, 26]. Thus, the process of leukoreduction before storage of blood in humans and dogs has promoted significant benefits in reducing the inflammatory response after blood transfusion, which consequently reduces the risk of FNHTR [26, 27].

Although we made the blood compatibility test between the donor and recipient, this was not enough to prevent the occurrence of transfusion reaction. Van Der Walt and Osterhoff [28] suggested that this test can detect only strong isoantibodies such as the anti-R in sheep and the anti-P in cattle.

Among the animals that presented hyperthermia (8 animals), 50% of then (two sheep from G0 and one from G15 and G30) presented acute reactions and 50% (two from G0 and one from G15 and G30) late reactions, and it is important to emphasize that the animals that showed increased temperature at *T*30 m continued to present this change for up to 96 hours. This differed from the late reactions, which were only observed at single observation times. Studies show that secondary alloimmunization can occur early between 24 and 48 hours after transfusion [29] or later, reaching its peak in seven to ten days of transfusion [30].

The animals that presented hyperthermia did not receive any medication and did not present temperature elevation above the normal range for this species. All the animals presented spontaneous remission of their hyperthermia.

With regard to individual heart rates, tachycardia occurred in all three groups. Although tachycardia is not in itself a transfusion reaction, when this sign is present it is important to investigate other variables such as blood pressure and pulmonary auscultation, in order to search for possible pulmonary edema. The commonest cause of tachycardia during or after blood transfusion is circulatory overload, due to administration of a large volume of blood or infusion at a high rate [8]. Withdrawal of a large volume of blood in order to induce anemia promoted activation of adaptive mechanisms such as increased heart and respiratory rates. However, after transfusion, some animals showed greater elevation of heart rate, probably due to the volume of blood received, given that the organism had adapted to a smaller volume of circulating blood. In such cases, the infusion rate cannot be held responsible for this, given that it was already low. Tachycardia can also occur in cases of circulatory overload, and this is usually associated with pulmonary edema. However, the animals in this study did not present this alteration. All the cases of tachycardia occurred within a 48-hour interval, thus indicating that in this case, there was an acute response to receiving blood.

At the baseline time, the mean values for heart rate were much higher than what would be considered normal for the species (70–90 bpm), in all three experimental groups [10], because of the reduction in blood cell volume and the number of red blood cells, resulting from induction of anemia. After the transfusion, although we saw that there had been a numerical reduction in this variable, statistical differences were only observed at *T*96 and *T*16 d in G0. This result can be explained by the great individual variability among the animals, especially given that the heart rate of some sheep increased in relation to *T*0, while most of the animals tended to present decrease in heart rate, thus giving rise to a high standard deviation. Reduction of heart rate relates to increased blood volume and displacement of fluid from the extravascular space to the intravascular space, in an attempt to maintain the organ's equilibrium [31, 32].

In the present study, there was no significant difference in respiratory rate between the groups. On the other hand, the large standard deviation that was seen particularly in G0 and G15 impaired the analysis (Figure 1). The hematologic data presented in Table 1 shows that the blood transfusion satisfactory increased the hemoglobin concentration and the number of circulating red blood cells (within the reference range for the species). Thus, the changes in heart and respiratory rates are not related to failure in the oxygen transport.

The significant increase in mean temperature at *T*96 in G0 (Figure 1) reflects occurrences of hyperthermia suffered by three animals of this group at this time, which raised the mean temperature by 1.1°C in relation to the baseline.

## 5. Conclusion

Transfusion of fresh or stored total blood improved the blood volume, but transfusion reactions occurred, demonstrating that a single transfusion of fresh or stored blood can cause inflammatory and febrile nonhemolytic transfusion reactions in sheep.

## Figures and Tables

**Table 1:** Mean values and standard deviations of the packed cell volume (%), red blood cell (*µ*L), and total hemoglobin (g/dL) of sheep receiving transfusion of blood fresh (G0) or stored for 15 days (G15) or 35 days (G35).

	Times										
	Groups	*T*0	*T*30	*T*6 h	*T*12 h	*T*24 h	*T*48 h	*T*72 h	*T*96 h	*T*8 d	*T*16 d
Packed cell volume (%)	G0	17 ± 5^a^	25 ± 6^b^	28 ± 4^b^	29 ± 5^b^	28 ± 5^b^	28 ± 3^b^	25 ± 4^b^	26 ± 4^b^	24 ± 5^b^	28 ± 4^b^
	G15	17 ± 2^a^	22 ± 2^a^	23 ± 2^b^	25 ± 2^b^	24 ± 3^b^	23 ± 2^b^	23 ± 2^b^	23 ± 2^b^	25 ± 5^b^	28 ± 3^b^
	G35	16 ± 4^a^	24 ± 3^b^	25 ± 3^b^	23 ± 3^b^	24 ± 3^b^	25 ± 3^b^	23 ± 2^b^	21 ± 4^a^	22 ± 1^a^	24 ± 3^b^

Red blood cell (*µ*L)	G0	4.82 ± 1.4^a^	6.81 ± 1.3^b^	7.64 ± 0.7^b^	7.89 ± 0.9^b^	7.76 ± 1.1^b^	7.37 ± 0.9^b^	6.88 ± 0.9^b^	7.07 ± 0.9^Ab^	6.49 ± 1.1^b^	7.21 ± 0.5^b^
	G15	5.10 ± 1.0^a^	6.53 ± 1.1^b^	6.94 ± 1.2^b^	7.44 ± 0.7^b^	7.24 ± 1.3^b^	6.87 ± 1.0^b^	6.86 ± 1.3^b^	6.60 ± 1.2^Bb^	6.95 ± 1.9^b^	7.52 ± 1.4^b^
	G35	4.63 ± 1.3^a^	7.00 ± 1.2^b^	7.56 ± 1.1^b^	6.99 ± 1.1^b^	7.16 ± 0.7^b^	7.84 ± 0.8^b^	7.18 ± 0.8^b^	6.30 ± 1.4^Bb^	6.61 ± 0.8^b^	7.07 ± 1.2^b^

Hemoglobin (g/dL)	G0	6.38 ± 2.0^a^	8.61 ± 2.1^b^	9.63 ± 1.5^b^	10.0 ± 2.0^b^	9.53 ± 2.1^b^	9.23 ± 1.6^b^	8.28 ± 1.4^b^	9.00 ± 1.6^b^	8.28 ± 1.6^b^	9.9 ± 0.9^b^
	G15	5.73 ± 0.9^a^	7.36 ± 1.8^a^	8.01 ± 0.9^b^	8.31 ± 0.9^b^	8.35 ± 1.1^b^	7.91 ± 1.0^b^	8.00 ± 1.4^b^	7.55 ± 0.9^b^	8.08 ± 1.8^b^	9.25 ± 0.4^b^
	G35	4.88 ± 1.2^a^	7.43 ± 0.7^b^	8.30 ± 1.4^b^	7.60 ± 1.1^b^	7.96 ± 0.8^b^	9.66 ± 1.1^b^	8.85 ± 1.0^b^	8.16 ± 1.3^b^	8.48 ± 0.5^b^	9.46 ± 1.3^b^

Different capital letters in columns mean difference between groups, while different small letters in the lines mean difference in relation to the baseline (*P* < 0.05).

**Table 2:** Clinical observation in sheep during transfusion with fresh or stored for 15 or 35 days whole blood (number of animals that present the symptom/total number of sheep).

Clinical manifestation	G0	G15	G35
Micturition	5/6	6/6	4/6
Defecation	1/6	5/6	2/6
Tachypnea	—	1/6	2/6
Fasciculation	—	1/6	1/6
Sudoresis	—	—	1/6
Bloating	—	—	1/6
Ejaculation	—	—	1/6

## References

[1] Harrell K., Parrow J., Kristensen A. (1997). Canine transfusion reactions. Part II. Prevention and treatment. *Compendium on Continuing Education for the Practicing Veterinarian*.

[2] Gonτalves S. (2006). *Reações transfusionais após a administração de concentrados de plaquetas em cães [Tese de Doutorado]*.

[3] Chin-Yee I., Arya N., D'Almeida M. S. (1997). The red cell storage lesion and its implication for transfusion. *Transfusion and Apheresis Science*.

[4] Sousa R. S., Barrêto-Júnior R. A., Sousa I. K. F., Chaves D. F., Soares H. S., Barros I. O., Minervino A. H. H., Ortolani E. L. (2013). Evaluation of hematologic, Blood gas, and select biochemical variables in ovine whole blood stored in CPDA-1 bags. *Veterinary Clinical Pathology*.

[5] Zallen G., Offner P. J., Moore E. E. (1999). Age of transfused blood is an independent risk factor for postinjury multiple organ failure. *The American Journal of Surgery*.

[6] Leal-Noval S. R., Jara-López I., García-Garmendia J. L., Marín-Niebla A., Herruzo-Avilés A., Camacho-Laraña P., Loscertales J. (2003). Influence of erythrocyte concentrate storage time on postsurgical morbidity in cardiac surgery patients. *Anesthesiology*.

[7] Hunt E., Moore J. S. (1990). Use of blood and blood products. *The Veterinary Clinics of North America: Food Animal Practice*.

[8] Hunt E., Wood B. (1999). Use of blood and blood products. *Veterinary Clinics of North America: Food Animal Practice*.

[9] Heddle N. M. (1999). Pathophysiology of febrile nonhemolytic transfusion reactions. *Current Opinion in Hematology*.

[10] Radostits O. M., Gay C. C., Hinchcliff K. W., Constable P. D. (2007). *Veterinary Medicine: A Textbook of the Diseases of Cattle, Sheep, Goats, Pigs and Horses*.

[11] Couto C. G., Nelson R. W., Couto C. G. (1998). Anemia. *Small Animal Internal Medicine*.

[12] Pugh D. G. (2004). *Clínica de Ovinos e Caprinos*.

[13] Bitterman H., Brod V., Weisz G., Kushnir D., Bitterman N. (1996). Effects of oxygen on regional hemodynamics in hemorrhagic shock. *The American Journal of Physiology*.

[14] Silva M. R., Figueiredo L. F. P., Younes R. N., Birolini D. (1999). Fisiopatologia do choque hipovolêmico. *Bases fisiopatológicas da cirurgia*.

[15] Reece W. O. (2006). *Dukes-Fisiologia dos Animais Domésticos*.

[16] Divers T. J. (2005). Blood component transfusions. *Veterinary Clinics of North America—Food Animal Practice*.

[17] Sharma A. D., Sreeram G., Erb T., Grocott H. P., Slaughter T. F. (2000). Leukocyte-reduced blood transfusions: perioperative indications, adverse effects, and cost analysis. *Anesthesia and Analgesia*.

[18] Chambers L. A., Kruskall M. S., Pacini D. G., Donovan L. M. (1990). Febrile reactions after platelet transfusion: the effect of single versus multiple donors. *Transfusion*.

[19] Brubaker D. B. (1990). Clinical significance of white cell antibodies in febrile nonhemolytic transfusion reactions. *Transfusion*.

[20] Balcomb C., Foster D. (2014). Update on the use of blood and blood products in ruminants. *Veterinary Clinics of North America: Food Animal Practice*.

[21] Perrotta P. L., Snyder E. L. (2001). Non-infectious complications of transfusion therapy. *Blood Reviews*.

[22] Eder A. F., Chambers L. A. (2007). Noninfectious complications of blood transfusion. *Archives of Pathology & Laboratory Medicine*.

[23] Tizard I. R. (2002). Imunidade inata: Inflamação. *Imunologia Veterinária-uma Introdução*.

[24] Novaretti M. C. Z., Bordin J. O., Langhi D. M., Covas D. T. (2007). Investigação Laboratorial em Pacientes com Anticorpos Eritrocitários. *Hemoterapia: Fundamentos e Prática*.

[25] Nielsen H. J., Reimert C., Pedersen A. N. (1997). Leucocyte-derived bioactive substances in fresh frozen plasma. *British Journal of Anaesthesia*.

[26] McMichael M. A., Smith S. A., Galligan A., Swanson K. S., Fan T. M. (2010). Effect of leukoreduction on transfusion-induced inflammation in dogs. *Journal of Veterinary Internal Medicine*.

[27] Federowicz I., Barrett B. B., Andersen J. W., Urashima M., Popovsky M. A., Anderson K. C. (1996). Characterization of reactions after transfusion of cellular blood components that are white cell reduced before storage. *Transfusion*.

[28] Van Der Walt K., Osterhoff D. R. (1969). Blood transfusion in cattle with special reference to the influence of blood groups. I: single transfusions into young animals and pregnant cows. *Journal of the South African Veterinary Medical Association*.

[29] Melo L., Bordin J. O., Langhi Júnior D. M., Covas D. T. (2007). Testes de Compatibilidade Sanguínea. *Hemoterapia: Fundamentos e Prática*.

[30] Thakral B., Saluja K., Sharma R. R., Marwaha N. (2008). Red cell alloimmunization in a transfused patient population: a study from a tertiary care hospital in north India. *Hematology*.

[31] Raiser A. G., Rabelo R. C., Crowe D. T. (2005). Choque. *Fundamentos de Terapia Intensiva Veterinária em Pequenos Animais*.

[32] Sousa R. S., Chaves D. F., Barrêto-Júnior R. A., Sousa I. K. F., Soares H. S., Barros I. O., Minervino A. H. H., Ortolani E. L. (2012). Clinical, haematological and biochemical responses of sheep undergoing autologous blood transfusion. *BMC Veterinary Research*.

